# P02.164. The effects of five sessions of cupping massage on chronic non-specific neck pain: a randomized controlled pilot study

**DOI:** 10.1186/1472-6882-12-S1-P220

**Published:** 2012-06-12

**Authors:** S Schumann, R Lauche, G Irmisch, C Hohmann, R Rolke, J Saha, H Cramer, K Choi, J Langhorst, T Rampp, G Dobos, F Musial

**Affiliations:** 1 Kliniken Essen-Mitte, Department for Internal and Integrative Medicine, Essen, Germany; 2 Universitätsklinikum Bonn, Klinik für Palliativmedizin, Bonn, Germany; 3 University of Tromsø, Department of Community Medicine, NAFKAM, Tromsø, Norway

## Purpose

To investigate whether five treatment sessions of cupping massage (CM) improve chronic non-specific neck pain (cNP) and influence pain related neurophysiologic measures.

## Methods

50 patients with cNP (age 53.3 ± 10.4 yrs) were examined at baseline and then randomized to treatment (TG) or standard medical care (SMC). Data were collected at baseline (T1) and T2 (for TG after treatment). TG received five treatment sessions of CM, while the SMC group was waiting. Subjective outcome measures included visual analogue scale for pain (VAS), Neck Pain Disability Index (NDI), and quality of life (SF36). Neurophysiologic testing included Quantitative Sensory Testing subtests mechanical detection threshold (MDT), pressure pain threshold (PPT), vibration threshold (VDT) and two-point discrimination (2PD) at the point of maximum pain (Pmax), close to maximum pain (Pclose), and hand and foot as control areas. ANCOVA analyses were conducted across post-pre differences with baseline values as covariates.

## Results

CM reduced pain (TG: pre 52.41 ± 20.9 vs post 29.94 ± 22.9; SMC: pre 45.89 ± 13.74 vs post 42.84 ± 15.83; p=.037) and NDI scores in TG, while SMC showed no change (TG: pre 14.47 ± 3.9 vs post 10.53 ± 3.7; SMC: pre 13.29 ± 6.1 vs post 13.35 ± 5.4; p=.003). “Physical health” (SF36) increased (TG: pre 35.35 ± 14.2 vs post 54.18 ± 19.8; SMC: pre 41.95 ± 14.24 vs post: 41.89 ± 14.01; p=.002). Neurophysiological data: TG showed an increase for PPT at Pmax (TG: pre 2.41 ± 0.2; post 2.45 ± 0.2; SMC: pre 2.43 ± 0.19 vs post 2.35 ± 0.24; p=.027).

## Conclusion

CM improved pain and increased subjective well being in cNP patients. The increase in PPT is consistent with a reduced peripheral sensitization of deeper tissues and supports previous findings on dry cupping in chronic cNP (Lauche et al., 2011).

